# Melanocytes in regenerative medicine applications and disease modeling

**DOI:** 10.1186/s12967-024-05113-x

**Published:** 2024-04-08

**Authors:** Kelly Coutant, Brice Magne, Karel Ferland, Aurélie Fuentes-Rodriguez, Olivier Chancy, Andrew Mitchell, Lucie Germain, Solange Landreville

**Affiliations:** 1 Department of Ophthalmology and Otorhinolaryngology-Cervico-Facial Surgery, Faculty of Medicine, Université Laval, Quebec City, QC Canada; 2 Regenerative Medicine Division, CHU de Québec-Université Laval Research Centre, Quebec City, QC Canada; 3 Centre de recherche en organogénèse expérimentale de l’Université Laval/LOEX, Quebec City, QC Canada; 4 Université Laval Cancer Research Center, Quebec City, QC Canada; 5 Department of Surgery, Faculty of Medicine, Université Laval, Quebec City, QC Canada

**Keywords:** Melanocytes, Regenerative medicine, Spheroid, Tissue engineering, Extracellular vesicles, Stem cells, Therapy, Disease modeling, Drug testing

## Abstract

Melanocytes are dendritic cells localized in skin, eyes, hair follicles, ears, heart and central nervous system. They are characterized by the presence of melanosomes enriched in melanin which are responsible for skin, eye and hair pigmentation. They also have different functions in photoprotection, immunity and sound perception. Melanocyte dysfunction can cause pigmentary disorders, hearing and vision impairments or increased cancer susceptibility. This review focuses on the role of melanocytes in homeostasis and disease, before discussing their potential in regenerative medicine applications, such as for disease modeling, drug testing or therapy development using stem cell technologies, tissue engineering and extracellular vesicles.

**Supplementary Information:**

The online version contains supplementary material available at 10.1186/s12967-024-05113-x.

## Introduction

Melanocytes are dendritic cells responsible for tissue pigmentation through melanogenesis, a biochemical process enabling melanin production within specialized organelles called melanosomes. During vertebrate development, melanocytes originate from the neural crest and later distribute across several organs, including the skin, eyes, hair follicles, ears, heart and central nervous system (reviewed in [1, 2]) [3]. Although their role in photoprotection in external organs is well characterized, the role of melanocytes in internal organs remains largely unknown or elusive. However, there is growing evidence that melanocytes also play a role in immunoregulation, hearing, vision, and tissue homeostasis (reviewed in [2, 4]).

The disruption of melanocyte functions can be caused by various pigmentary conditions, in which external organs are either hypopigmented, like in vitiligo, oculocutaneous albinism and Waardenburg syndrome (WS); or hyperpigmented, like in lentigo senilis or Café-au-lait macules (reviewed in [5–7]) [8, 9]. Some pigmentary disorders can also result in hearing or vision impairments. Moreover, skin, ocular or mucosal melanomas originate from transformed melanocytes. Diseases associated with melanocyte depletion are not painful but result in a greater sensitivity to ultraviolet (UV) radiation, exposing them to higher risk of developing skin cancers (reviewed in [10]).

Currently, there are no curative therapies for most pigmentary disorders. However, significant progress in the field of regenerative medicine has opened exciting new avenues for the development of alternative disease models, drug testing systems and treatments. Several cutting-edge technologies and fields of study such as tissue-engineered substitutes, genome editing with CRISPR-Cas9, induced pluripotent stem cells (iPSCs), spheroids and extracellular vesicles (EVs) now prove to be indispensable for modeling pigmentary diseases and hold great promises for future therapies.

In this review, we focus on the use of melanocytes in regenerative medicine applications, such as for disease modeling, drug testing and therapy. To fully understand the potential of melanocytes in regenerative medicine, we first describe their origin, functions, and associated pathologies, and then explore the newest and future approaches to integrate these unique pigmented cells in regenerative medicine.

## Melanocytes in homeostasis and disease

### Origin of melanocytes

**Fig. 1:**
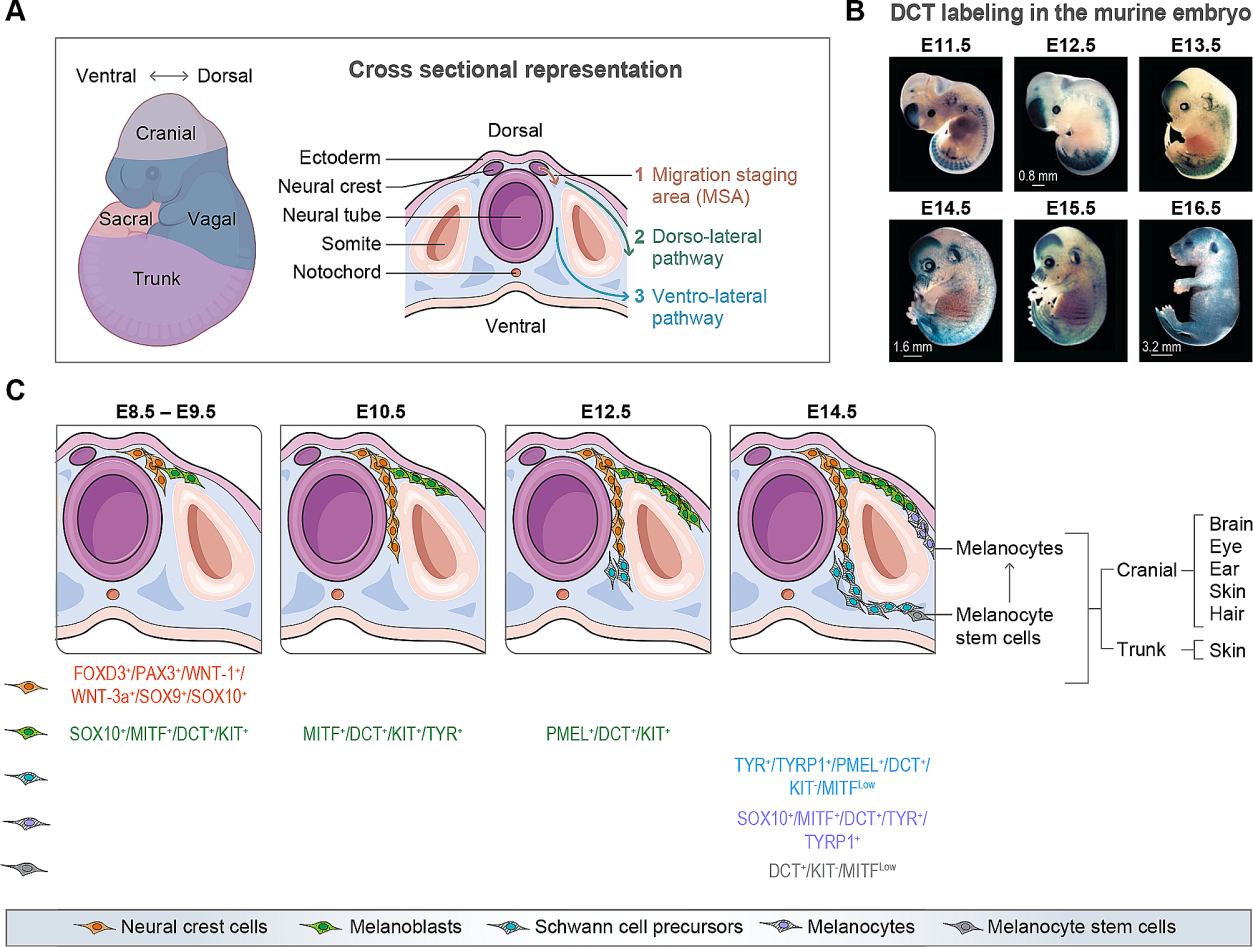
Embryonic development of melanocytes (**A**) Schematic and cross-sectional representation of the neural crest subpopulations in a mouse embryo (E10.5). Neural crest cells present in the MSA migrate primarily along the dorso-lateral pathway where they differentiate to become melanoblasts and ultimately mature melanocytes. (**B**) DCT labeling of melanoblasts and melanocytes in the murine embryo during development (E11.5 to E16.5; modified from [26]). Until E13.5, DCT positive cells (in blue: *Dct-lacZ*) are primarily localized dorsally, before colonizing to the ventral axis. The presence of melanoblasts in hair follicles can be seen at E15.5-E16.5. Scale bars: 0.8 mm (E12.5), 1.6 mm (E14.5) and 3.2 mm (E16.5). (**C**) Progression of melanocyte lineage during murine embryonic development from stages E.8.5 to E14.5. Melanocytes are derived from the cranial and trunk neural crest populations. Neural crest cells present in the MSA migrate along the dorso-lateral pathway (E8.5-E9.5) where they differentiate into melanocytes (E14.5). In parallel, once the dorso-lateral pathway is fully developed (E12.5), SCPs will migrate along the ventro-lateral pathway where they differentiate into McSCs (E14.5) that will later become melanocytes

The neural crest, originating from the ectoderm during gastrulation, is a transient embryonic structure present in all vertebrates (reviewed in [11]). It comprises four regions: cranial, trunk, vagal and sacral neural crests (Fig. 1A) (reviewed in [12]). Cranial neural crest cells engender melanocytes found in eyes, ears, hair follicles and face skin, whereas other cutaneous melanocytes arise from the trunk neural crest (reviewed in [13]). Similarly, heart melanocytes are derived from the vagal neural crest (reviewed in [14]), meningeal melanocytes from the cranial neural crest or from Schwann cell precursors (SCPs), and melanocytes found in mucous membranes come from both cranial and trunk neural crests [15, 16]. During neurulation, the neural folds converge to create the neural tube. This spatial reorganization allows the delamination of neural crest cells derived from the neural folds [17]. These multipotent cells are precursors to a variety of cell types, including Schwann cells and melanoblasts, which are the precursors of melanocytes (reviewed in [18, 19]) [20, 21]. Neural crest cells start in the migration staging area (MSA), then undergo an epithelial-mesenchymal transition before migrating along either the dorso-lateral pathway (between the mesoderm and ectoderm) or the ventro-lateral pathway (between the dermomyotome and the ectoderm) (Fig. 1A and C) (reviewed in [19]) [17, 22]. This tightly regulated migration of neural crest cells ultimately defines the location of their cell progeny (reviewed in [23]).

In humans, the migration of melanoblasts is not thoroughly characterized, but studies in mice have provided significant insights. Neural crest cells differentiate into melanoblasts at mid-gestation in the MSA, beginning proliferation at embryonic day 8.5 (E8.5). By E9, melanoblasts are primarily localized in the head and face, with fewer in cervical and trunk regions (Fig. 1B) [24–26]. At E10.5, they enter the dorso-lateral pathway and begin to differentiate into melanocytes, a process regulated by WNT signaling [27]. The microphthalmia-associated transcription factor (MITF), activated by WNT-1 and WNT-3a, regulates genes crucial for melanoblast-to-melanocyte differentiation. These include tyrosinase (TYR), tyrosinase-related protein 1 (TYRP1), melanoma antigen recognized by T cells 1 (MART-1; also known as Melan-A), dopachrome tautomerase (DCT; also known as TYRP2) and premelanosome protein (PMEL; also known as PMEL17, SILV or GP100) (reviewed in [28]) [24, 27, 29]. Around E12.5, SCPs enter the ventro-lateral pathway [20]. They are the main source of skin melanocytes, as well as the non-cutaneous melanocytes in the heart, inner ear and meninges [17]. Interestingly, melanocytes share common molecular hallmarks with Schwann cells, such as SRY-box 10 (SOX10) and DCT [20]. The relationship between these types is evident as Schwann cells can transdifferentiate into melanocytes after losing contact with an axon (reviewed in [12]). From E12/E13, melanoblasts can be found in the mitral and tricuspid valves of the heart, and in various structures of the inner ear [3, 30]. In human embryos, melanoblasts surround the otic vesicles and neural tube by week 5, indicating migration toward the ear [30]. Melanocytes also reach brain meninges in mice, rats and humans, and are present in human buccal mucosa from 20 weeks of gestation (reviewed in [31]) [32–34]. At E15.5, melanoblasts reside in the hair follicle bulge, where some transition into melanocyte stem cells (McSCs) [25]. Other melanoblasts migrate towards the follicle bulb, maturing into melanocytes by transiently expressing c-KIT [35]. In the eye, melanoblasts are observed in the choroid mesenchyme at E15.5 in mice, while pigmented melanocytes are observed in the human choroid at week 27 [25, 36].

### Functions of melanocytes

Melanocytes are crucial for skin and eye photoprotection through melanin production, distribution and accumulation, and may have diverse functions in internal organs (e.g., inner ears, heart, meninges and mucosa). Melanin acts as an antioxidant and a UV radiation absorber, preventing skin and eye aging and diseases such as age-related macular degeneration (AMD; OMIM#603075) (reviewed in [37, 38]) [39]. Also, melanocyte loss-of-function in the iris and retinal pigment epithelium (RPE) is linked to photoreceptor damage and vision defects (reviewed in [40]). Furthermore, melanin expression in the brain helps mitigate nitric oxide-induced neurotoxicity (reviewed in [41]). Mammals express two types of melanin pigments: the brownish black eumelanin and the reddish yellow pheomelanin (reviewed in [41]). While eumelanin is photoprotective, pheomelanin synthesis generates reactive oxygen species (ROS), potentially increasing the risk of skin cancers like melanoma (OMIM#155600). In fact, a high pheomelanin-to-melanin ratio is associated with the development of skin cancers (reviewed in [41]) [42, 43].

Melanocytes contribute to immunity by expressing major histocompatibility complex class II molecules and presenting antigens (reviewed in [4]) [44]. They also secrete several cytokines like interleukin (IL)-1α, IL-1β, IL-8, and transforming growth factor (TGF)-β1 in the skin, and attract immune cells through chemokines such as chemokine (C-X-C motif) ligand (CXCL)-8 to -11, chemokine (C-C motif) ligand (CCL)-2 and CCL-5 in the choroid (reviewed in [12]) [17, 45]. Human choroidal melanocytes also express toll-like receptors (TLRs) which are involved in pathogen clearance and inflammation in the eye (reviewed in [46]) [47, 48].

In addition to their roles in photoprotection, attenuation of ROS damage, and immunity, melanocytes are critical in the development, maintenance, and proper functioning of various tissues. Melanocytes are vital for cochlear development, contributing to the secretion of the endolymph and maintaining the endocochlear potential necessary for hearing [49]. Their absence can lead to deafness, congenital hearing loss or auditory-pigmentary syndrome (reviewed in [2]). In the heart, melanocytes of the tricuspid valve leaflet participate in cardiac tissue stiffness, which ultimately affects the viscoelastic properties of the heart [50]. Although the functions of mucosal and meningeal melanocytes are not well established, a decrease of melanocytes in meninges of older mice suggests a potential role of these cells in brain ageing [16].

### Cell signaling within melanocytes

The MITF isoform M (MITF-M) is the master regulator of melanocyte proliferation, survival and development, as well as melanin synthesis (melanogenesis) (reviewed in [51]) [9, 52]. In healthy melanocytes, MITF induces the transcription of enzymes implicated in melanogenesis such as TYR, TYRP1 and DCT, but also of melanosome-related proteins including PMEL and G-protein-coupled receptor 143 (GPR-143) [29, 53–56]. Pathological mutations in the MITF gene are associated with familial and sporadic melanomas, but also with rare genetic disorders (OMIM#193510/#103500) including the Waardenburg syndrome type-2 A, characterized by hearing loss and pigmentation defects (reviewed in [51]) [9].

The expression of MITF is regulated by other transcription factors such as cyclic adenosine monophosphate (cAMP) responsive element-binding protein (CREB), paired box gene 3 (PAX3) which is mutated in WS type-1 and − 3 (OMIM#193500/#148820), SOX transcription factors 9 (SOX9) or 10 (SOX10), the latter being mutated in WS type-2E and − 4 C (OMIM#611584/#613266) (reviewed in [57, 58]) [59–61]. Many signaling pathways regulate the expression of these transcription factors (see Supplementary Table S1), some of which are detailed below.

### The melanocortin 1 receptor (MC1R) signaling pathway

Proopiomelanocortin (POMC) is a neuropeptide secreted by the anterior pituitary gland [62]. Keratinocytes and melanocytes also secrete POMC, which is increased by UV radiation [62–64]. POMC cleavage results in the production of α-melanocyte-stimulating hormone (α-MSH), which in turn binds to the MC1R, a GPCR expressed in melanocytes (reviewed in [65]). The binding of α-MSH to MC1R causes an increase of intracellular cAMP, that activates CREB to trigger MITF expression (reviewed in [66]). Non-pathological polymorphisms in the MC1R gene are frequent and cause skin and hair pigmentation variations across humans and animals (OMIM#266300) (reviewed in [66]).

### The opsin (OPN) signaling pathway

OPNs are light-sensitive GPCRs expressed by photoreceptor cells in the eye but also by melanocytes and keratinocytes in the skin [67]. OPNs are activated by visible light (OPN1-SW (blue-violet), OPN2 (green), OPN3 (violet-green)) or UV (OPN5) (reviewed in [68]). OPN3 and OPN5 activation increases calcium fluxes in melanocytes, activating calmodulin-dependent protein kinase II (CAMKII), an inducer of CREB signaling [69, 70]. OPN3 also activates the MAPK signaling pathway, leading to the gene transcription of TYR and DCT [70]. Finally, OPN5 activation triggers the PKC signaling pathway and leads to the gene transcription of TYR, TYRP1 and DCT [69, 70].

### Melanosome biogenesis and function

**Fig. 2:**
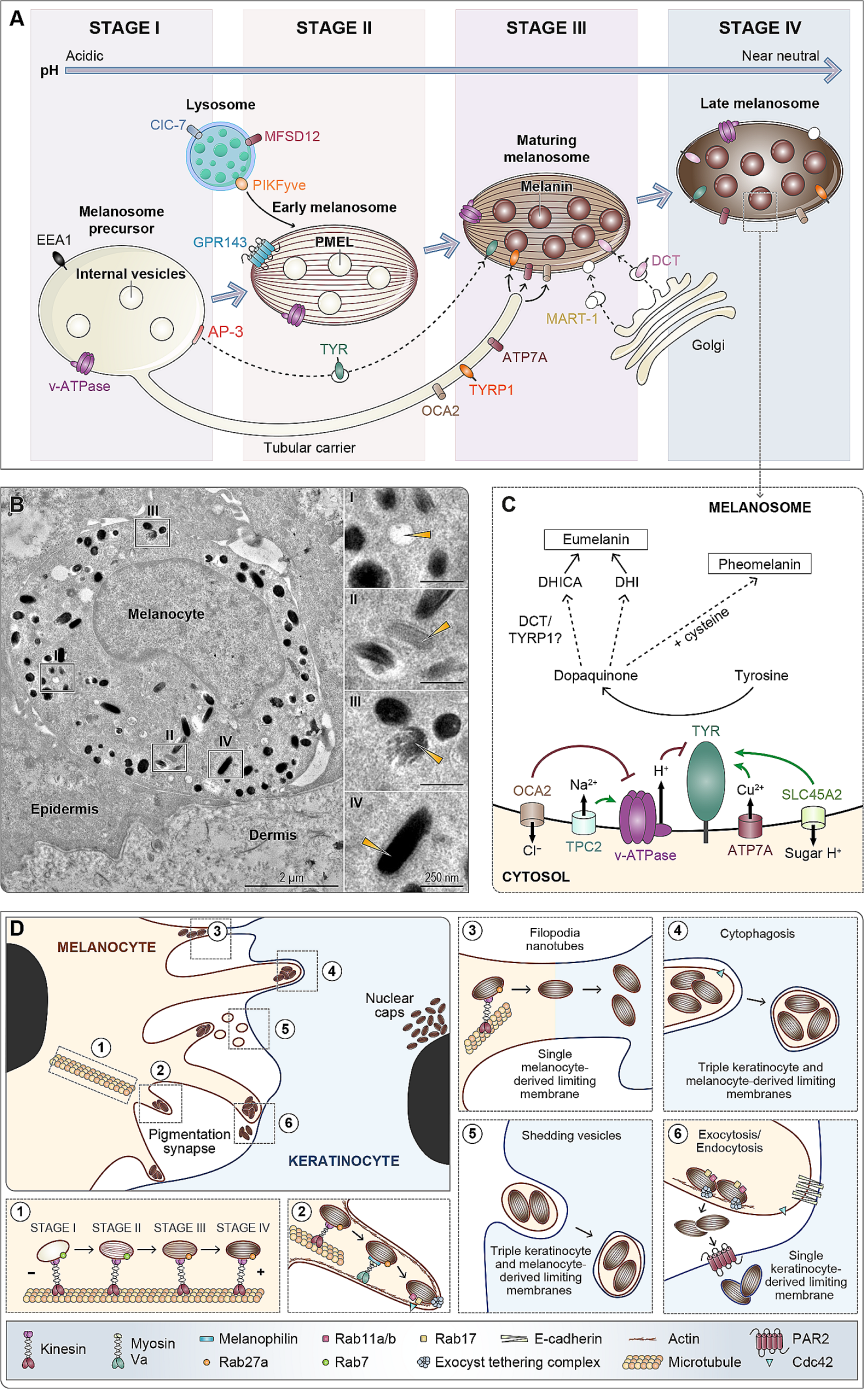
Melanosome biogenesis, structure, functions and fate. (**A**) Schematic of melanosome biogenesis and maturation pathways. Melanogenic cargoes such as OCA2, MART-1 or TYR are transferred to maturing melanosomes through diverse trafficking pathways during melanogenesis. Melanosome maturation is influenced by pH and membrane contacts with other organelles including lysosomes. (**B**) Transmission electron micrograph of a melanocyte within the skin epidermis. The insets highlight examples of melanosomes at the different stages of maturation. PMEL fibrils are visible in stages II and III melanosomes before being buried under accumulating melanin pigments in stage IV melanosomes. (**C**) Schematic of the eumelanin and pheomelanin biosynthetic pathways in melanosomes. Several enzymes such as TYR, TYRP1 and DCT, and transporters including vATPase, TPC2 and ATP7A regulate melanogenesis through modulation of pH, membrane potential or ion content within melanosomes. (**D**) Schematic of the putative melanin transfer mechanisms occurring within the skin epidermis. Melanosomes are transported by kinesins across microtubules toward melanocyte dendrite tips (**inset 1**). Stage IV melanosomes are then trapped within actin fibers by a tripartite complex composed of Myosin Va, Melanophilin and Rab27a (**inset 2**). Melanin transfer could then result from filopodia nanotube formation (**inset 3**), cytophagosis of melanocyte dendrites (**inset 4**), vesicle transfer (**inset 5**) or exocytosis/endocytosis (**inset 6**). Within keratinocytes, melanin forms nuclear caps above cell nuclei to protect DNA from photodamage

Melanosome biogenesis and maturation is a multi-stage process (Fig. 2A), which can be observed by transmission electron microscopy (Fig. 2B). Stage I melanosomes are similar to early endosomes, and mostly express early endosomal markers, such as early endosome antigen 1 (EEA1) [71]. The differentiation pathway from stage I to stage II melanosomes is not yet fully understood. Possible mechanisms include: (i) expression of melanocyte-specific intracellular GPR143 [72], which is defective in patients with type-1 ocular albinism (OMIM#300500), ii) involvement of specific endosomal subsets, which have been shown to fulfil specific functions in other cells [73], or iii) fusion between lysosomes and early melanosomes, possibly via the activity of the lipid kinase PIKfyve [74].

Melanosome maturation relies on three major membrane trafficking pathways that deliver melanogenic cargoes and ion transporters to stages III and IV melanosomes (Fig. 2A). One essential enzyme for melanin synthesis, TYR, is sorted into AP-3-dependent vesicles from early endosomes to maturing melanosomes [75]. Other cargoes such as MART-1 and DCT travel by vesicles from the trans-Golgi network to maturing melanosomes [76]. Most other melanogenic cargoes, such as copper transporter ATP7A, pink-eyed dilution protein homolog 2 (OCA2) and TYRP1, are sorted into early endosomal tubules that are pulled along microtubules by molecular motors [77, 78]. Mutations in proteins that are necessary for the biosynthetic delivery of melanogenic cargoes or ion transporters to maturing melanosomes lead to oculocutaneous albinism (OCA), and are observed in patients with Hermansky-Pudlak (HPS), Griscelli (GS) and Chediak-Higashi (CHS) syndromes (reviewed in [79, 80]).

### Melanin synthesis (melanogenesis)

Both eumelanin and pheomelanin pigments are produced from dopaquinone, a reaction product resulting from the catalysis of tyrosine by TYR [81]. In the presence of cysteine, dopaquinone undergoes a succession of redox reactions leading to the synthesis of pheomelanin. By contrast, in the absence of cysteine, DCT and TYRP1 help convert dopaquinone into a series of intermediates that give rise to eumelanin (reviewed in [82]). Loss-of-function mutations in TYR cause OCA1A or 1B (OMIM#203100/#606952), the most severe forms of OCA, due to the complete depletion of both pheomelanin and eumelanin (reviewed in [5]). Mutations in DCT trigger OCA8 (OMIM#619165), a milder form of OCA, in which patients synthesize eumelanin through DCT-independent reactions, leading to more oxidative stress and melanocyte apoptosis [83, 84]. Unlike TYR and DCT, the function of TYRP1 is unclear. Mutations in TYRP1 cause OCA3 (OMIM#203290), a mild form of OCA that is characterized by diluted pigmentation [85, 86]. However, whether TYRP1 has an enzymatic role, a chaperone activity or an antioxidant function in melanogenesis is still debated (reviewed in [18]).

### Regulation of melanogenesis

Melanosomal pH plays a key role in melanogenesis. Early-stage melanosomes are highly acidic due to the activity of the proton-pumping vacuolar H^+^ ATPase (v-ATPase) [87], to promote PMEL assembly into amyloid-like fibril sheets that give melanosomes their ovoid shape (Fig. 2B) [71, 87–90]. However, in later stages of melanosome biogenesis, pH must reach 6.8 to allow optimal TYR activity and melanin synthesis [91, 92].

At least three ion transporters regulate melanosomal pH: OCA2 and solute carrier family 45 member 2 (SLC45A2), which neutralize melanosomal pH and promote TYR activity; and the two-pore channel 2 (TPC2), which acidifies melanosomes and reduces TYR activity (Fig. 2C) [93–95]. OCA2 and TPC2 are anion and cation extruder channels, respectively, that modulate v-ATPase activity through regulation of melanosomal membrane potential [94, 96]. SLC45A2 is a symporter channel that expels sugars and protons from the melanosomal lumen to the cytosol [97]. While OCA2 and SCL45A2 mutations cause type-2 and type-4 OCA respectively (OMIM#203200/#606574), TPC2 polymorphisms are responsible for non-pathogenic pigment variations (OMIM#612267) [98–100].

Other determinants, including ion content and organelle crosstalk, also regulate melanogenesis (Fig. 2A and C). Expression of the copper transporter ATP7A in maturing melanosomes is crucial for melanogenesis as copper is a known cofactor for TYR activity [101]. Loss-of-function mutations of this transporter in patients with Menkes disease (MNK; OMIM#309400) results in hypopigmentation [102]. Two lysosomal or late endosomal proteins, the chloride-proton exchanger ClC-7 and the major facilitator superfamily domain containing 12 (MFSD12), also appear to regulate pheomelanin production in melanocytes [103, 104]. Calcium transporters expressed in non-melanosomal compartments could also influence melanogenesis, including transient receptor potential cation channel subfamily M member 1 (TRPM1), SLC24A4 and SLC24A5 [105–107]; mutations of the latter are involved in OCA6 (OMIM#113750).

### Melanin transfer and tissue pigmentation

Unlike uveal melanocytes and RPE cells, which synthesize melanin almost entirely before birth and retain it in their cytoplasm throughout life, skin melanocytes constantly produce and transfer pigments to neighboring keratinocytes (Fig. 2D) (reviewed in [1]). In the skin, melanocytes reside in the basal layer of the epidermis and extend their dendrites to contact up to 36 keratinocytes and form the so-called epidermal-melanin unit [111]. Melanin transfer from skin melanocytes to keratinocytes is orchestrated by a complex series of events in both cell types. Mature melanosomes, expressing small Rab GTPase Rab27a [108], are transported to the ends of melanocyte dendrite tips by kinesin, a molecular motor protein (Fig. 2D, inset 1) [109]. They are then captured into the actin cytoskeleton network by a tripartite complex composed of myosin Va, melanophilin and Rab27a [110, 111]. Melanosome positioning on the actin cytoskeleton is critical for melanin transfer since this process is impeded by cytochalasin B, an actin polymerization inhibitor [112]. Other components of the Rab GTPase protein superfamily including Rab11a, Rab11b, and Rab17, as well as the exocyst tethering complex and Cdc42, also appear to be involved in melanin transfer (Fig. 2D, inset 2) [113–117].

The precise mechanism involved in the transfer of melanosomes from melanocytes to keratinocytes is subject to controversy. In vitro co-culture systems using human or animal cells have shown that melanosomes could be transferred, either by filopodia nanotubes, cytophagocytosis or shedding vesicles (Fig. 2D, insets 3–5) [116, 118, 119]. According to these models, transferred pigments should be wrapped in their original melanosomal membrane, as well as within a melanocyte and/or keratinocyte-derived plasma membrane. However, these predicted double- or triple-membrane compartments are rarely observed by transmission electron microscopy [117, 120, 121]. Moreover, TYRP1, a transmembrane protein expressed at the melanosomal membrane in melanocytes, is not detected in keratinocytes that incorporate melanin [122]. These discrepancies indicate that the melanosomal membranes are either rapidly degraded once internalized by keratinocytes, or another transfer mechanism is at play.

Most recently, in situ transmission electron microscopy experiments have shown that melanin transfer could occur through an exocytosis/endocytosis mechanism (Fig. 2D, inset 6) [117, 121]. In this model, mature melanosomes fuse with the plasma membrane of the melanocyte and release melanin, referred to as melanocores, into the extracellular space. Melanocores could then be endocytosed within single membrane compartments by neighboring keratinocytes that are in contact with melanocyte dendrites. To support this model, recent evidence has shown that melanocores are more easily internalized than melanosomes by keratinocytes, through the protease-activated receptor 2 (PAR2), whose specific expression in keratinocytes is increased upon UVB exposure [122, 123]. Moreover, keratinocyte-melanocyte adhesion sites, referred to as pigmentation synapses, could facilitate melanocore endocytosis and appear to be crucial for melanin transfer. Dark-skinned individuals with Darier disease (DAR; OMIM#124200), a genodermatosis caused by mutations in the ATP2A2 gene altering cell adhesion through defects in calcium signaling, can sometimes exhibit hypopigmented macules (reviewed in [124]). Calcium-dependent E-cadherin-mediated adhesion within pigmentation synapses also plays an important role in melanin transfer [125].

Once melanin is transferred to keratinocytes, it forms parasol-like structures above cell nuclei, known as nuclear caps [126]. Although a causative link between photoprotection and nuclear cap positioning and content has yet to be established, early evidence suggests that the quantity of supranuclear melanin correlates with less DNA damage in normal human skin samples [127]. The mechanism of melanin positioning and nuclear cap formation is not well understood. However, patients with type-1 Dowling-Degos disease (DDD1; OMIM#179850), a genodermatosis caused by mutations in the keratin 5 (KRT5) gene, possess keratinocytes that incorporate melanin pigments but lack nuclear caps, suggesting a role for KRT5 in melanin positioning [128].

## Melanocytes in biomedical research

**Fig. 3:**
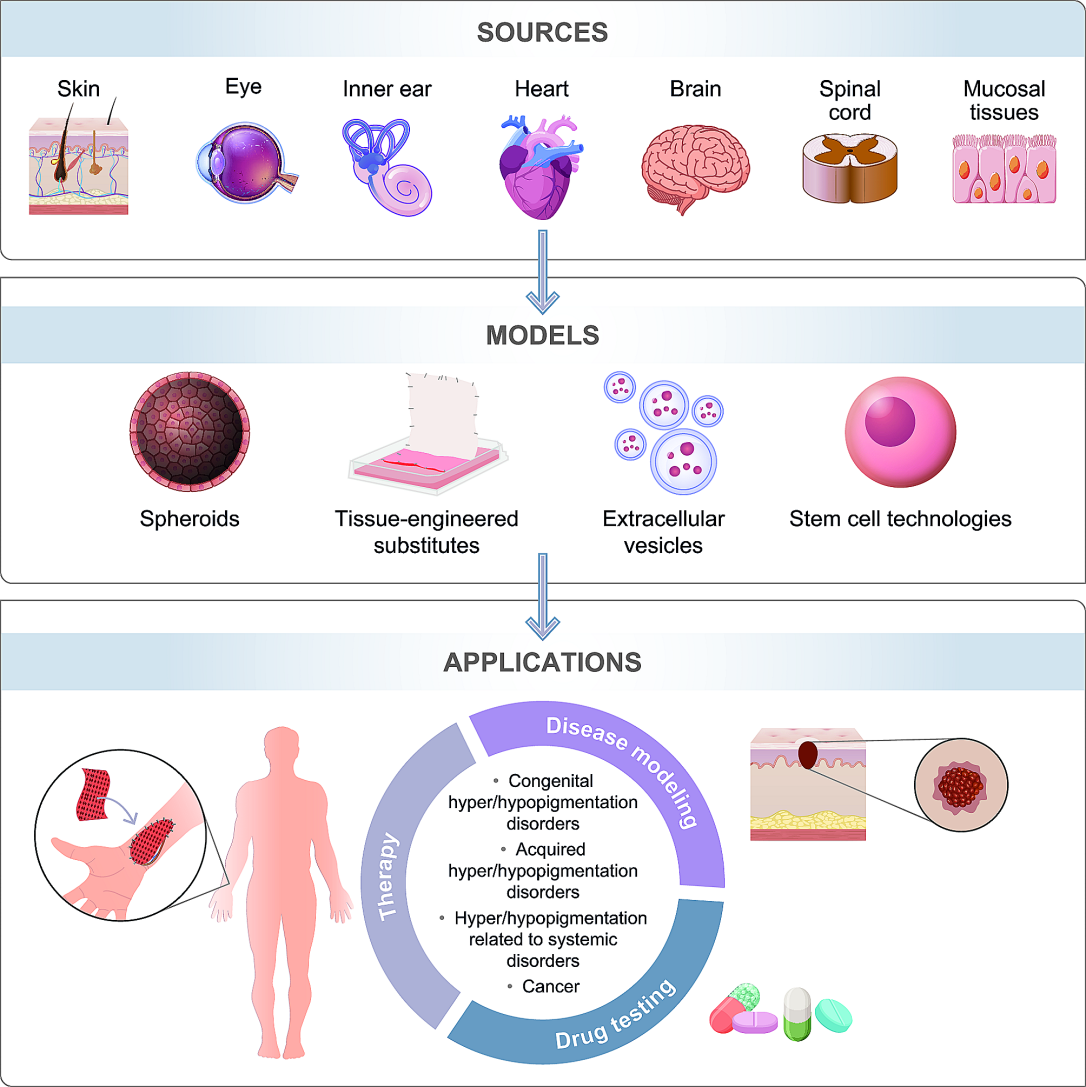
Sources, research models and potential applications of melanocytes in regenerative medicine. Melanocytes are distributed not only in the skin and eye, but also in other organs/tissues such as the inner ear, heart, meninges of the brain and spinal cord, and mucosal tissues. The research models used to study their potential in regenerative medicine include tissue-engineered substitutes, spheroids, extracellular vesicles and patient-derived iPSCs. These models have promising clinical applications such as disease modeling, drug testing and therapy development which will enhance our understanding and treatment of pigmentation disorders and melanomas

Treating pigmentary disorders presents a set of clinical challenges due to their multifaceted or unidentified etiologies. Nevertheless, these disorders pose a significant burden for patients, who often suffer from hypopigmentation of the skin, eyes and hair, may have increased susceptibility to UV-induced skin cancers, and sometimes also present various disabilities such as hearing and vision loss, or neurodegeneration (reviewed in [10]). In addition to lacking sufficient photoprotection, patients with pigmentary disorders also often experience psychological distress, low self-esteem, and social exclusion. A better understanding of pathophysiological mechanisms involved in the development of pigmentary disorders and melanomas is needed to improve patients’ treatments. Over the past few decades, several regenerative medicine applications have emerged to help identify, target, or prevent the development of these pathologies (Fig. 3). In this section, we will provide an updated overview of these recent advances.

### Melanocytes in disease modeling

#### Stem cell technologies

Melanocytes generated from patient-derived induced pluripotent stem cells (iPSCs) and embryonic stem cells (ESCs) have been valuable tools to model diseases. Through the application of these techniques, fully differentiated human iPSC-derived melanocytes (hiMels) have been generated using several growth factors, such as WNT3A, stem cell factor and bone morphogenetic protein 4 (BMP4) [129, 130]. In another model, mesenchymal stromal cells capable of differentiating in neural crest lineage cells, specifically melanocyte precursors, were obtained from ESCs treated with small molecule compounds such as glycogen synthase kinase 3 beta (GSK3β) and TGF-β inhibitors in a defined basal medium [131]. These models allowed to study skin disorders where HPS and CHS patient-derived hiMels have been shown to produce melanosomes with altered structural characteristics, impacting both melanin production and transfer, and have thus helped understand the pathogenesis of these genetic disorders [21]. HiMels derived from café-au-lait macules have also been used to explore the role of neurofibromin 1 (NF1) in melanocyte differentiation [132]. The loss of NF1 was shown to induce senescence during differentiation into melanocytes, demonstrating the importance of this protein in melanocyte lineage commitment and pigmentation [132].

Native human McSCs found in hair follicles are also highly valuable for disease modeling, potentially being implicated in the development of melanomas. Indeed, a subset of melanoma cells expressing CD20 was able to differentiate across multiple cell lineages, self-renew and induce tumors when grafted into mice. These findings suggest a potential role for stem cells in the development of this type of cancer [133]. In agreement with this hypothesis, it has recently been shown that melanoma-prone McSCs can be activated through long exposure to UVB or through natural stem cell cycling induced by depilation, leading to the formation of cutaneous melanoma (CM) and gives rise to invasive epidermal melanomas in mice [134, 135]. Although the mechanisms underlying the transformation of McSCs into cancer cells are not yet fully understood, several studies indicate various gene susceptibilities [136, 137].

### Genome editing

Numerous studies are exploring innovative uses of CRISPR/Cas9 technology in the field of melanocyte research. These technologies allow the study of melanocyte biology and its role in unique organs and tissues which may provide insights into novel therapeutic strategies. Some studies using CRISPR have generated TYR knockout (KO) melanocytes, demonstrating a significant association between melanin and lipofuscin production [138]. Notably, post-UV radiation, the prevalence of lipofuscin granules was markedly pronounced in TYR-KO cells compared to their wildtype counterparts [138]. In zebrafish, one study demonstrated the augmented efficiency of synthetic, chemically modified guide RNAs when combined with recombinant Cas9 protein for genome editing [139]. This enhanced technique successfully knocked in a diverse set of markers, including bacterial nitroreductase. A promising development in zebrafish genome editing was identified with zLOST, utilizing long single-stranded DNA templates for improved homology-directed repair (HDR), even restoring pigmentation in specific albino embryos [140].

In research on pigmentation and albinism, an iPSC line was developed, incorporating two key TYR gene variants, providing a robust in vitro model to explore albinism mechanisms [141]. New insights into oculocutaneous albinism type 1 were gleaned by identifying two novel TYR gene variants in a Chinese hypopigmented patient, offering potential therapeutic avenues through CRISPR technology [142].

Cancer research has also benefited from CRISPR technology advancements. In CM, the knockout of cyclin-dependent kinase 2 (CDK2) resulted in G0/G1 phase arrest, instigating early apoptosis of melanoma cells [143]. Melanoma-specific enhancers in the MET gene, driven by the MITF transcription factor, have been identified, opening avenues to address drug resistance in cancer treatments [144]. In a parallel study, the targeting of acid ceramidase (AC) in melanoma cells demonstrated its significant involvement in tumor progression, suggesting a potential therapeutic intervention for advanced melanoma [145]. Another revelation in melanoma research highlighted the potential of bromodomain inhibitors (BETi), emphasizing the critical role of the amphoterin-induced gene and open reading frame 2-protein tyrosine kinase 7 (AMIGO2-PTK7) pathway in their therapeutic impact [146].

Diverse studies further revealed adenosine deaminase acting on RNA 1 (ADAR1)’s significant role in neural crest development and its impact on melanocytes and Schwann cells [147]. Moreover, insights into the KIT gene’s role in Yorkshire pigs’ pigmentation and erythropoiesis were uncovered, offering novel perspectives on gene mutations [148].

### Tissue-engineering and spheroid modeling

Although 2D culture systems have helped explore melanocyte function and melanoma treatment response, they do not mimic the 3D environment of native tissues which is known to play important physiological and pathophysiological roles [149]. For example, a study has shown that melanin synthesis is widely repressed in 2D culture systems, while it is favored in 3D spheroid models [150]. Skin tissue engineering also represents a good model for understanding the mechanisms implicated in pigmentation in both homeostasis and disease states. For example, UV irradiation of pigmented bilayered tissue-engineered skin substitutes (P-TESSs) can help understand the mechanisms underlying tanning and skin disease progression, such as how UV exposure damages DNA in cutaneous cells, and the associated repercussions at the cellular and tissue levels [151]. Tissue engineering can also be used to study ocular conditions, such as AMD, a leading cause of blindness in industrialized countries that affects the posterior segment of the eye (reviewed in [152, 153]). A 3D model of the RPE/choroid segment was established using choroidal melanocytes, fibroblasts, and endothelial cells as well as RPE cells isolated from human donor eyeballs [154]. Such a model could provide a better understanding of the intercellular communication between choroidal melanocytes, endothelial cells and RPE to develop new therapies for AMD.

CM is a highly metastatic cancer that arises from skin melanocytes. In The Cancer Genome Atlas (TCGA), CM is categorized into four subtypes based on activating gene mutations: *BRAF* mutant, *NRAS/KRAS/HRAS* mutant, *NF1* mutant and triple-wildtype cases [155]. Spheroids have been used as models to understand the treatment resistance of CM. The 3D culture of CM cells in poly-hydroxyethylmethacrylate-coated plates changes the expression of a large variety of transcripts when compared to gene expression profiles of cells cultured in 2D in presence or absence of collagen [156]. These include the CXCL family, IL8, CCL20, ANGPT4 or CD49, as well as the response to heat stress, TGF-β and IL-3/IL-4 signaling [156]. The 3D state and cell population heterogeneity in spheroids induce a change in drug response to BRAF and MEK inhibitors in CM (reviewed in [157, 158]) [159]. Spheroid models allowed the comparison of the genetic profile of BRAF^V600^-inhibitor resistant cells to sensitive cell counterparts; some of the most upregulated genes were SPC25, CCL2, CCNE2 and PLK1, all known to be involved in the metastatic process [159].

Another way of studying the development and progression of CM is through the use of reconstructed 3D environments mimicking skin properties and cell types of normal human skin, also referred to as tissue-engineered skin subsitutes (TESSs). Melanoma cells have also been integrated in tissue-engineered constructs to study invasion and metastasis [160–164]. TESSs have been used to show that melanoma cells expressing the fibroblast growth factor-2 (FGF-2) proliferate at a higher rate and are more invasive than those not expressing FGF-2 [164]. CM has also been studied with more complex TESS models that incorporate blood and lymphatic capillaries [160]. Adding these components to TESSs more accurately mimics the melanoma biology, providing an opportunity to study complex processes such as angiogenesis and to test new drugs [160, 161]. In their study, Vörsmann and colleagues used both reconstructed skin and spheroids made with melanoma cell lines with different genetic backgrounds to recreate the melanoma microenvironment [165]. Such a model could represent a useful tool to test drugs in vitro, as they incorporate not only cancerous cells but the associated microenvironment components found in melanoma tumors as well.

Uveal melanoma (UM), developing from melanocytes of the choroid, the iris or the ciliary body, is a rare but deadly cancer with 50% of patients affected by metastasis in various sites, but primarily the liver (reviewed in [166]). UM is classified in four molecularly distinct subtypes based on chromosomal abnormalities and gene mutations according to TCGA [167]. In the case of UM, spheroids were mostly generated to study the capacity of cells to form such structures [168, 169]. Spheroids have also been used as tool to analyze the different responses to doxorubicin (chemotherapeutic agent) and selumetinib (MEK inhibitor) treatments using various established cell lines such as OMM2.5, MM66, MP41 or 92.1, thus linking different genetic profiles to different resistance properties [165, 170]. As an alternative to established cell lines, primary tumor cells obtained following an enucleation, the second most common procedure to treat UM, can be used to realize such tests [165, 170]. The reconstructed choroidal niche described earlier can also be used to study in vitro the modifications in the tumor microenvironment during the growth of choroidal melanoma (reviewed in [171]) [154].

### Extracellular vesicles (EVs)

The Minimal Information for Studies of Extracellular Vesicles (MISEV) guidelines describe EVs as secreted cell particles that are delimited by a lipid bilayer with no functional nucleus and no replicative abilities (reviewed in [172]). They are found in all biological fluids such as blood or urine, and also in vitro in the culture medium conditioned by cells (reviewed in [173]). EVs are rich biological cargoes, consisting of proteins, nucleic acids, microRNAs, metabolites and lipids (reviewed in [172, 173]). Because they are important for cell communication and signaling, EVs are central actors in the development of diverse pathologies, including pigmentary disorders and melanomas. Interestingly, proteins associated with melanosome biogenesis such as Rab GTPases (RABs), tetraspanin CD63, SNAP receptors (SNAREs), and Biogenesis of Lysosome-related Organelle Complexes (BLOCs) are also involved in the biosynthesis, transport and release of EVs (reviewed in [174, 175]).

UVB-irradiated melanocytes have been shown to release more fibronectin-loaded EVs, an EV subset known to reduce apoptotic bodies and cell death, therefore playing an important role in cell survival during UV-exposure [176]. The transcriptome analysis of keratinocytes exposed to EVs derived from UVA-irradiated melanocytes reveals the activation of TGF-β and IL-6/STAT3 signaling pathways and an upregulation of miR-21, which induce keratinocyte proliferation and migration [177].

In CM, the biological cargo of EVs differed between healthy and pathological melanocytes. Proteomic analyses of CM-EVs demonstrated an enrichment of proteins including p120-catenin, radixin, annexins A1/A2, syntenin, hyaluronan and proteoglycan link protein 1 (HAPLN1) involved in angiogenesis, cell invasion, migration and metastasis in melanoma (reviewed in [178]) [179–182]. EVs are known to be involved in pre-metastatic niche formation (reviewed in [183]) [184, 185]. CM-derived EVs participate in the cell phenotype transition in the tumor microenvironment by targeting fibroblasts that switch to cancer-associated fibroblasts (CAFs) [186, 187]. They have been shown to contain miR-211, miR-155 and miR-210 that target the MAPK pathway and aerobic glycolysis, and decrease oxidative phosphorylation in fibroblasts [186, 187]. A pro-inflammatory role of EVs derived from CM cells has been observed compared to normal melanocytes (reviewed in [188]).

UM cells secrete EVs that reach the liver and induce the release of cytokines and growth factors in hepatic cells, such as macrophage inhibitory factor (MIF), which in turn promotes the recruitment of melanoma cells to the liver in a murine model [189]. In comparison, EVs derived from epidermal melanocytes (non-cancerous control) contain a low level of MIF and do not activate cell signaling in hepatocytes in vitro [189]. EVs from normal choroidal melanocytes (NCMs) were recently isolated from conditioned culture medium and characterized as normal controls for UM studies [190, 191]. Although NCM-EVs were internalized by hepatic stellate cells, they did not increase their activation or contractile function in collagen gels, in contrast to cancer cells, which increased both properties [190]. In addition, EVs derived from NCMs contained a different cargo than UM-EVs, and were unable to induce the proliferation and malignant transformation of BRCA1-deficient fibroblasts after their internalization [191]. Tumoral EVs are thus facilitating melanoma progression by modeling the microenvironment and metastatic spreading, and the study of EVs from healthy melanocytes will help to understand how malignant cells profit from physiological cell-cell communication mechanisms.

## Melanocytes in therapy

### Stem cell technologies

Therapeutic approaches using McSCs or iPSCs can provide alternative treatments for pigmentary disorders. For example, the development of pigmentation in hair follicles of immunodeficient unpigmented mice was made possible up to seven weeks after the transplantation of hiMels, which were shown to localize to the basal layer of the hair bulb epithelium in vitro [192]. Similarly, pigment cells expressing MART-1 could be detected as early as 3 days after hiMels injection into immunodeficient unpigmented mice [193]. Overall, these studies suggest that iPSC-derived melanocytes could be a potential source for autologous transplantation to treat skin disorders such as vitiligo. The use of iPSCs could also potentially overcome the limitations of current melanocyte transplantation techniques, such as the limited availability of donor cells and the risk of immune rejection.

Native McSCs also have a therapeutic potential for long-lasting repigmentation of ageing tissues. Following genotoxic stresses (e.g., chemicals, drugs, or radiations), hair graying is thought to be due to the loss of McSCs resulting in the depletion of differentiated melanocytic progeny in the hair bulge (reviewed in [194, 195]). B-cell lymphoma 2 (BCL-2) protein and MITF deficiencies have also been shown to accelerate this process [196, 197]. A modulation of the McSC niche might thus prevent or reverse the loss of pigmentation in ageing skin and hair (reviewed in [194]).

Stem cells also serve as a reservoir for autologous melanocytes sources for cell-based therapies. It has been demonstrated that multipotent dermal stem cells obtained from human neonatal foreskins have the potential to differentiate into various cell types, including pigmented melanocytes [198].

### Cell inoculation and tissue-engineered substitute grafting

Cultured melanocytes have been used in several case report studies to improve skin pigmentation in patients suffering from vitiligo or piebaldism. In these studies, cultured autologous melanocytes extracted from healthy donor sites were used to treat unpigmented skin defects using suction or liquid-nitrogen blisters [199, 200]. In other case report studies, autologous suspensions of cultured melanocytes or co-cultures of keratinocytes and melanocytes were applied into the wound bed directly after the surgical removal of unpigmented skin areas in patients receiving skin grafts [8, 199, 200]. Using these techniques, skin pigmentation was generally shown to be well restored, but some areas, such as the hairline or fingers, proved more difficult to treat than others [200]. A refinement of this treatment could be achieved using 3D spheroids, as they improve the survival of melanocytes compared to cell suspensions [201]. It has already been shown in a preclinical study that transplanting chitosan-based melanocyte spheroid patch after preparing the recipient sites with psoralen and UVA-induced sunburn can facilitate melanocyte transplantation [202]. It would be interesting to see if difficult-to-repigment areas (such as hairlines and fingers) can be pigmented with this method.

Cultured epithelial autografts (CEAs), initially used for the treatment of severe burns, have also been tested to correct hypopigmentation defects in patients suffering from vitiligo and piebaldism [8, 203–205]. Pigmented CEAs have been generated from isolated autologous keratinocytes and melanocytes and grafted after the surgical removal of the hypopigmented skin areas [203, 204]. A 2-year follow-up study in six patients indicated that this treatment restored pigmentation in 88–100% of the grafted areas [204].

Although P-TESSs have not yet been used to treat patients with pigmentary disorders, this approach could be an interesting therapeutic avenue. Preclinical studies in athymic mice have indeed shown that P-TESSs can maintain a homogeneous pigmentation over time after grafting [151, 206]. Further analyses have also revealed that melanin produced in these P-TESSs can be efficiently transferred from melanocytes to keratinocytes, form nuclear caps above cell nuclei, and protect the skin substitutes from DNA damage [151, 207, 208].

### Extracellular vesicles (EVs)

EVs have been closely related to several pigmentation disorders such as vitiligo. In the skin, melanocytes interact with keratinocytes by direct contact or via secreted EVs. Keratinocyte-derived EVs that are internalized by melanocytes can regulate melanogenesis. For example, keratinocyte-derived EVs containing miR-330-5p have been shown to reduce melanin production and decrease TYR expression in melanocytes that incorporate these EVs [209].

EVs have been shown to mediate drug resistance to chemotherapeutic medications through different mechanisms (reviewed in [210]). Melanoma cells release EVs that are internalized in hepatic stellate cells, which leads to their activation and an increase of their pro-fibrogenic properties [190]. In addition, melanoma cells subjected to therapeutic agents have been shown to secrete more EVs that can modify macrophage phenotype favoring melanoma growth [211].

EVs may also play a role in the development of pathologies in skin. Human skin is directly in contact with external stimuli such as UV radiation. In response to these stimulations, melanocytes must counteract the cytotoxic effects that can generate pathologies such as melasma or CM via melanogenesis, so their survival is important to maintain skin pigmentation. To understand the pathogenesis, some studies focus on the response to UV radiation through EVs. UV radiation has also been found to have a direct impact on the crosstalk between melanocytes and keratinocytes [177, 212]. Studies have shown that UVA irradiation of melanocytes increased their release of EVs and the miRNA transport by EVs to keratinocytes [177, 212]. This release of miRNAs was associated with EV internalization by keratinocytes [212].

## Melanocytes in drug testing

The use of hiMels provides a more physiologically relevant system for drug screening than traditional cell culture models, and may improve the likelihood of identifying drugs that are effective in vivo. Using hiMels, screening assays have thus been developed for identifying drugs that promote or inhibit melanin production, in order to improve treatments of pigmentary disorders [213].

P-TESSs and spheroids have been used to test compounds intended for cosmetic purposes to investigate their mechanisms of action. For example, the treatment of P-TESSs with kynurenine, a metabolite associated with immune tolerance, was shown to inhibit melanogenesis [214]. The inhibitory effect of fucoxanthin on melanin production was confirmed using spheroids produced with skin melanocytes [215]. Recently, P-TESSs produced with hiMels provide a better model for in vitro experiments and could reduce animal testing (reviewed in [216]).

Spheroids produced from melanoma cells have been used to test several BRAF mutant inhibitors, to help identify genes implicated in metastatic outgrowth (reviewed in [158]). Models of melanoma progression have been developed combining P-TESSs and spheroids produced from melanoma cell lines with varying genetic backgrounds to investigate the effects of different drugs [165]. Complex models of P-TESSs encompassing a lymphatic system and/or a microvasculature may also prove useful to study drug diffusion through these structures and evaluate their effects on cancer cells [160, 161]. Lastly, UM has also been studied using spheroids with various established cell lines to screen the effect of various drugs on cell resistance and invasion patterns [170, 217].

## Discussion

Despite many decades of research in pigment cell biology, the roles and functions of melanocytes in health and disease are still not fully understood. While the molecular mechanisms driving melanogenesis in melanocytes have been well studied in various cell lines and animal models, the processes regulating their communication with other cell types and response to diverse environmental cues are only beginning to be uncovered. Therefore, novel study approaches involving tissue engineering, stem cell technologies, or extracellular vesicles will likely be instrumental to expand knowledge on melanocyte biology and pigmentary disorder pathogenesis.

This review describes the origin and functions of melanocytes and provides perspective on their potential in regenerative medicine from a basic, translational, or clinical research standpoint. In addition, this review also identifies some limitations. For example, in hyperpigmentation or hypopigmentation disorders, only certain parts of the body are affected, therefore, tools to restore pigment must target affected areas rather than act systemically. Caution must also be taken with procedures promoting melanocyte proliferation to make sure it does not induce melanoma. Conversely, the use of inhibitors against melanogenesis in cancer must spare healthy melanocytes.

To have a better understanding of melanocytes’ involvement in diseases, more research needs to be done to better characterize their physiology. For example, it is known that melanocytes transfer melanin to keratinocytes in the skin to protect against free radicals and oxidative stress, but this function is not demonstrated in melanocytes of other tissues. In addition, since melanocytes work closely with other cell types such as keratinocytes in the skin or RPE cells in the posterior segment of the eye, inhibition or induction of melanocyte proliferation can lead to adverse effects in the targeted tissue or surrounding tissues (reviewed in [218]) [121, 219, 220]).

Therapeutic approaches will also require more knowledge in melanocyte development and human models. Mutations can alter the expression of proteins in some ocular pathologies, and it can be difficult to recover the mutated cells in vitro. Advances in CRISPR technology could thus be used to correct diseased cells or incorporate mutations in melanocytes to produce models of frequent mutations found in pigmentary disorders or melanomas; more research is warranted in this area to identify therapeutic targets.

Over the past several years, EVs have garnered significant interest for eventual treatment of diseases, including cancers (reviewed in [221]). EVs are often associated with modifications of the microenvironment in distal tissues which become favorable to tumor development or metastasis. EVs could also be developed as drug or gene delivery tools. Nevertheless, questions remain. In vitro experiments lack a functional immune system. To inject EVs carrying drugs, it will be necessary to ensure that they are not destroyed by immune cells. For this, it will be preferable to isolate EVs directly from the patient, bringing new uncertainties such as the best EV source (blood or lymphatic fluid sampling), and the concentration to be recovered and injected. As EVs are vehicles secreted by all cell types, any potential treatment using “smart EVs” will have to target cells of interest with specific surface proteins.

The extensive use of animal models raises ethical concerns [222]. Fortunately, alternative in vitro models such as tissue-engineered substitutes and spheroids have been developed to better mimic the complexity of in vivo cell interactions in order to properly test drugs. Accordingly, intricate 3D architectures can be reproduced in vitro allowing the study of complex phenomena. These include: melanocyte dysfunction in pigmentary disorders, cell invasion and metastasis in the context of cutaneous and ocular melanomas, tissue grafting, and the development of relevant tools for drug evaluation (reviewed in [171, 223]) [154, 224, 225].

In summary, there is a growing interest in the use of melanocytes in regenerative medicine, and researchers of the field are currently developing different innovative tools or models (e.g., spheroids, tissue-engineered substitutes, extracellular vesicles, stem cell technologies) for improving pigmentary disease modeling and targeted therapies.

### Electronic supplementary material

Below is the link to the electronic supplementary material.

Supplementary Material 1

## Data Availability

Not applicable.

## Abbreviations

α-MSH: α-melanocyte-stimulating hormone
AC: acid ceramidase
ADAR1: adenosine deaminase acting on RNA 1
AMD: age-related macular degeneration
AMIGO2-PTK7: amphoterin-induced gene and open reading frame 2-protein tyrosine kinase 7
APCs: antigen presenting cells
BCL-2: B-cell lymphoma 2
BETi: bromodomain inhibitors
BLOCs: biogenesis of lysosome-related organelle complexes
BMP4: bone morphogenetic protein 4
c-AMP: cyclic adenosine monophosphate
CAFs: cancer-associated fibroblasts
CAMKII: calmodulin-dependent protein kinase II
CCL: chemokine (C-C motif) ligand
CDK2: cyclin-dependent kinase 2
CEAs: cultured epithelial autografts
CHS: Chediak-Higashi syndrome
CM: cutaneous melanoma
CREB: cAMP responsive element binding protein
CXCL: chemokine (C-X-C motif) ligand
DAR: Darier disease
DCT: dopachrome tautomerase
DDD1: type-1 Dowling-Degos disease
DHI: 5,6-dihydroxyindole
DHICA: 5,6-dihydroxyindole-2-carboxylic acid
EEA1: early endosome antigen 1
ESCs: embryonic stem cells
EVs: extracellular vesicles
FGF-2: fibroblast growth factor-2
GPCR: G protein-coupled receptor
GPR-143: G-protein-coupled receptor 143
GS: Griscelli syndrome
GSK3β: glycogen synthase kinase 3 beta
HAPLN1: hyaluronan and proteoglycan link protein 1
HDR: homology-directed repair
hiMels: human iPSC-derived melanocytes
HPS: Hermansky-Pudlak syndrome
IL: interleukin
iPSCs: induced pluripotent stem cells
KO: knockout
KRT5: keratin 5
MAPK: mitogen activated protein kinase
MART1: melanoma antigen recognized by T cells 1
MC1R: melanocortin 1 receptor
McSCs: melanocyte stem cells
MFSD12: major facilitator superfamily domain containing 12
MIF: macrophage inhibitory factor
MISEV: minimal information for studies of extracellular vesicles
MITF: microphthalmia-associated transcription factor
MITF-M: MITF isoform M
MNK: Menkes disease
MSA: migration staging area
NCMs: normal choroidal melanocytes
NF1: neurofibromin 1
OCA: oculocutaneous albinism
OPN: opsin
P-TESSs: pigmented bilayered tissue-engineered skin substitutes
PAR2: protease-activated receptor 2
PMEL: premelanosome protein
POMC: proopiomelanocortin
RABs: Rab GTPases
ROS: reactive oxygen species
RPE: retinal pigment epithelium
SCPs: Schwann cell precursors
SLC45A2: solute carrier family 45 member 2
SNAREs: SNAP receptors
SOX: SRY-box
TCGA: The Cancer Genome Atlas
TESSs: tissue-engineered skin substitutes
TGF: transforming growth factor
TLRs: toll-like receptors
TPC2: two pore channel 2
TRPM1: transient receptor potential cation channel subfamily M member 1
TYR: tyrosinase
TYRP1: tyrosinase-related protein 1
UM: uveal melanoma
UV: ultraviolet
v-ATPase: vacuolar H^+^ ATPase
WS: Waardenburg syndrome
