# Supplementary material for: Melanocytes in regenerative medicine applications and disease modeling
Source: J Transl Med. 2024 Apr 8;22:336. doi: 10.1186/s12967-024-05113-x (PMC11003097; doi:10.1186/s12967-024-05113-x)
Supplement: Supplementary file 1 — Supplementary Material 1 [file 12967_2024_5113_MOESM1_ESM.docx]

**Table S1. Signaling pathways in melanocytes.**

Description of signaling pathways, biological relevance and pathological implication in melanocytes.

| **Receptors** | **Extracellular ligands** | **Secreting cell types** | **Signaling pathways** | **Melanocyte-specific transcription factors** | **Factor involved in development** | **Biological processes** | | | | | **Associated pigmentary disorders (OMIM#)** | **References** |
| --- | --- | --- | --- | --- | --- | --- | --- | --- | --- | --- | --- | --- |
|  |  |  |  |  |  | Proliferation | Migration | Survival | Dendricity | Melanogenesis |  |  |
| Bone morphogenetic protein receptor (BMPR) | Bone morphogenetic protein 4 (BMP-4) | Keratinocyte, melanocyte | MAPK | MITF (↓) | No |  |  |  |  | ↓ | Unknown | [1] |
|  | Bone morphogenetic protein 6 (BMP-6) | Keratinocyte, melanocyte | MAPK | MITF (↑) | No |  |  |  |  | ↑ | Unknown | [1] |
| c-KIT | Stem cell factor (SCF) | Fibroblast, keratinocyte | MAPK | MITF (↑) | Yes |  |  |  |  | ↑ | c-KIT deletion in piebaldism (172800) SCF mutation in WS-2F (619947) | [2, 3] |
| Endothelin receptor B (EDNRB) | Endothelin-1 (ET-1) | Keratinocyte, endothelial cells | PKC | MITF (↑) | Yes | ↑ | ↑ |  | ↑ | ↑ | EDNRB mutation in WS-4A (277580) | [4-7] |
|  | Endothelin-2 (ET-2) | Endothelial cells | PKC | MITF (↑) | Yes | ↑ |  |  |  | ↑ | EDNRB mutation in WS-4A (277580) | [6, 8] |
|  | Endothelin-3 (ET-3) | Endothelial cells | PKC | MITF (↑) | Yes | ↑ | ↑ |  |  | ↑ | EDNRB mutation in WS-4A (277580) ET-3 mutation in WS-4B (613265) | [6, 9-11] |
| Erb-B2 receptor tyrosine kinases 3 and 4 (erbB3, erbB4) | Neuregulin 1 (NRG1) | Fibroblast | MAPK | SOX10 (↑) | Yes | ↑ | ↑ |  |  |  | Decreased NRG1 expression in vitiligo | [12-15] |
| Fibroblast growth factor receptor (FGFR) | Basic fibroblast growth factor (bFGF) | Keratinocyte, fibroblast | MAPK | MITF (↑) | No | ↑ | ↑ | ↑ |  | ↑ | Decreased FGF-2 expression in vitiligo | [16-18] |
| Frizzleds (FZDs) | WNT1 | Unknown | β-catenin | MITF (↑) | Yes |  |  |  |  | ↑ | Unknown | [19] |
|  | WNT3A | Unknown | β-catenin | MITF (↑) | Yes |  |  |  |  | ↑ | Unknown | [20] |
| Growth macrophage-colony stimulating factor receptor (GMCSFR) | Growth macrophage-colony stimulating factor (GM-CSF) | Keratinocyte | MAPK | MITF (↑) | No | ↑ |  |  |  | ↑ | Decreased GM-CSF expression in vitiligo | [21-23] |
| Interferon gamma receptor (IFNGR) | Interferon gamma (IFN-ɣ) | T cell | JAK1/STAT1 | MITF (↓) | No |  |  | ↓ |  | ↓ | Increased IFN-ɣ expression in vitiligo | [24, 25] |
| Interleukin-1 receptor (IL1R) | Interleukin-1 alpha (IL-1α) | Keratinocyte, Langerhans cell | Unknown | MITF (↓) | No | ↓ |  |  |  | ↓ | Unknown | [26, 27] |
| Interleukin-4 receptor (IL4R) | Interleukin-4 | T cell | MAPK | MITF (↓) | No |  |  |  |  | ↓ | Unknown | [28] |
| Interleukin-6 receptor (IL6R) | Interleukin-6 (IL-6) | Keratinocyte, Langerhans cell, adipose derived stem cells | Unknown | MITF (↓) | No | ↓ |  |  |  | ↓ | Unknown | [27, 29] |
| Keratinocyte growth factor receptor (KGFR) | Keratinocyte growth factor (KGF) | Fibroblast | MAPK | Unknown | No |  |  |  |  | ↑ | Unknown | [30] |
| Kremen 1 and 2 (Krm1, Krm2); Liprotein receptor-related protein 6 (LRP6) | Dickkopf related protein 1 (DKK1) | Fibroblast | β-catenin | MITF (↓) | No | ↓ |  |  |  | ↓ | Unknown | [31] |
| Leukemia inhibitory factor receptor (LIFR); Glycoprotein 130 (gp130) | Leukemia inhibitory factor (LIF) | Keratinocyte | MAPK | MITF (↑) | No | ↑ |  |  |  | ↑ | Unknown | [32] |
| Melanocortin receptor 1 (MC1R) | Alpha-melanocyte stimulating hormone (α-MSH) | Keratinocyte, melanocyte (obtained from POMC cleavage) | cAMP | CREB (↑) MITF (↑) SOX9 (↑) | No | ↑ |  |  |  | ↑ | MC1R mutation in OCA2 (203200) | [33-36] |
|  | Adrenocorticotropic hormone (ACTH) | Keratinocyte, melanocyte (obtained from POMC cleavage) | cAMP | MITF (↑) | No | ↑ |  |  |  | ↑ | MC1R mutation in OCA2 (203200) | [37, 38] |
|  | Agouti signaling protein (ASP) | Keratinocyte (dermal papilla) | cAMP | MITF (↓) SOX9 (↓) | No |  |  |  |  | ↓ | MC1R mutation in OCA2 (203200) | [35, 39] |
| Melanocortin receptor 4 (MC4R) | Beta-melanocyte stimulating hormone (β-MSH) | Keratinocyte, melanocyte (POMC) | cAMP | MITF (↑) | No |  |  |  |  | ↑ | Unknown | [40] |
| MET proto-oncogene, receptor tyrosine kinase | Hepatocyte growth factor (HGF) | Fibroblast | MAPK | Unknown | No | ↑ |  |  |  |  | Unknown | [41, 42] |
| Prostaglandin E2 receptor 4 (EP4) | Prostanglandin E2 (PGE2) | Keratinocyte | cAMP | Unknown | No | ↑ |  |  |  | ↑ | Unknown | [43] |
| Prostaglandin F2-alpha receptor (FP receptor) | Prostaglandin F2alpha (PGF2α) | Keratinocyte | PKC | Unknown | No |  |  |  | ↑ | ↑ | Unknown | [44] |
| Transforming growth factor beta receptor 2 (TGFbR2) | Transforming growth factor β (TGF-β) | Keratinocyte | SMAD2 | PAX3 (↓) MITF (↓) | No |  |  |  |  | ↓ | Unknown | [45, 46] |
| Tumor necrosis factor receptor (TNFR) | Tumor necrosis factor alpha (TNF-α) | Keratinocyte, Langerhans cell, melanocyte | Unknown | MITF (↓) | No | ↓ |  |  |  | ↓ | Unknown | [27, 47] |

**SUPPLEMENTARY REFERENCES**

1. Singh SK, Abbas WA, Tobin DJ. Bone morphogenetic proteins differentially regulate pigmentation in human skin cells. J Cell Sci. 2012;125(Pt 18):4306-19.

2. Fleischman RA, Saltman DL, Stastny V, Zneimer S. Deletion of the c-kit protooncogene in the human developmental defect piebald trait. Proc Natl Acad Sci U S A. 1991;88(23):10885-9.

3. Grabbe J, Welker P, Dippel E, Czarnetzki BM. Stem cell factor, a novel cutaneous growth factor for mast cells and melanocytes. Arch Dermatol Res. 1994;287(1):78-84.

4. Hara M, Yaar M, Gilchrest BA. Endothelin-1 of keratinocyte origin is a mediator of melanocyte dendricity. J Invest Dermatol. 1995;105(6):744-8.

5. Kadono S, Manaka I, Kawashima M, Kobayashi T, Imokawa G. The role of the epidermal endothelin cascade in the hyperpigmentation mechanism of lentigo senilis. J Invest Dermatol. 2001;116(4):571-7.

6. Regazzetti C, De Donatis GM, Ghorbel HH, Cardot-Leccia N, Ambrosetti D, Bahadoran P, et al. Endothelial Cells Promote Pigmentation through Endothelin Receptor B Activation. J Invest Dermatol. 2015;135(12):3096-104.

7. Yada Y, Higuchi K, Imokawa G. Effects of endothelins on signal transduction and proliferation in human melanocytes. J Biol Chem. 1991;266(27):18352-7.

8. Hou L, Pavan WJ, Shin MK, Arnheiter H. Cell-autonomous and cell non-autonomous signaling through endothelin receptor B during melanocyte development. Development. 2004;131(14):3239-47.

9. Garcia RJ, Ittah A, Mirabal S, Figueroa J, Lopez L, Glick AB, et al. Endothelin 3 induces skin pigmentation in a keratin-driven inducible mouse model. J Invest Dermatol. 2008;128(1):131-42.

10. Hofstra RM, Osinga J, Tan-Sindhunata G, Wu Y, Kamsteeg EJ, Stulp RP, et al. A homozygous mutation in the endothelin-3 gene associated with a combined Waardenburg type 2 and Hirschsprung phenotype (Shah-Waardenburg syndrome). Nat Genet. 1996;12(4):445-7.

11. Lee HO, Levorse JM, Shin MK. The endothelin receptor-B is required for the migration of neural crest-derived melanocyte and enteric neuron precursors. Dev Biol. 2003;259(1):162-75.

12. Choi W, Wolber R, Gerwat W, Mann T, Batzer J, Smuda C, et al. The fibroblast-derived paracrine factor neuregulin-1 has a novel role in regulating the constitutive color and melanocyte function in human skin. J Cell Sci. 2010;123(Pt 18):3102-11.

13. Gordon-Thomson C, Jones J, Mason RS, Moore GP. ErbB receptors mediate both migratory and proliferative activities in human melanocytes and melanoma cells. Melanoma Res. 2005;15(1):21-8.

14. Paratore C, Goerich DE, Suter U, Wegner M, Sommer L. Survival and glial fate acquisition of neural crest cells are regulated by an interplay between the transcription factor Sox10 and extrinsic combinatorial signaling. Development. 2001;128(20):3949-61.

15. Rani S, Kumari U, Bhardwaj S, Parsad D, Sharma VL, Kumar R. Decreased expression of neuregulin1 in the lesional skin of vitiligo patients. Int J Dermatol. 2019;58(2):242-9.

16. Halaban R, Langdon R, Birchall N, Cuono C, Baird A, Scott G, et al. Basic fibroblast growth factor from human keratinocytes is a natural mitogen for melanocytes. J Cell Biol. 1988;107(4):1611-9.

17. Puri N, van der Weel MB, de Wit FS, Asghar SS, Das PK, Ramaiah A, et al. Basic fibroblast growth factor promotes melanin synthesis by melanocytes. Arch Dermatol Res. 1996;288(10):633-5.

18. Wu CS, Lan CC, Chiou MH, Yu HS. Basic fibroblast growth factor promotes melanocyte migration via increased expression of p125(FAK) on melanocytes. Acta Derm Venereol. 2006;86(6):498-502.

19. Dunn KJ, Williams BO, Li Y, Pavan WJ. Neural crest-directed gene transfer demonstrates Wnt1 role in melanocyte expansion and differentiation during mouse development. Proc Natl Acad Sci U S A. 2000;97(18):10050-5.

20. Guo H, Xing Y, Liu Y, Luo Y, Deng F, Yang T, et al. Wnt/beta-catenin signaling pathway activates melanocyte stem cells in vitro and in vivo. J Dermatol Sci. 2016;83(1):45-51.

21. Hirobe T, Furuya R, Hara E, Horii I, Tsunenaga M, Ifuku O. Granulocyte-macrophage colony-stimulating factor (GM-CSF) controls the proliferation and differentiation of mouse epidermal melanocytes from pigmented spots induced by ultraviolet radiation B. Pigment Cell Res. 2004;17(3):230-40.

22. Imokawa G, Yada Y, Kimura M, Morisaki N. Granulocyte/macrophage colony-stimulating factor is an intrinsic keratinocyte-derived growth factor for human melanocytes in UVA-induced melanosis. Biochem J. 1996;313 ( Pt 2)(Pt 2):625-31.

23. Moretti S, Spallanzani A, Amato L, Hautmann G, Gallerani I, Fabiani M, et al. New insights into the pathogenesis of vitiligo: imbalance of epidermal cytokines at sites of lesions. Pigment Cell Res. 2002;15(2):87-92.

24. Natarajan VT, Ganju P, Singh A, Vijayan V, Kirty K, Yadav S, et al. IFN-gamma signaling maintains skin pigmentation homeostasis through regulation of melanosome maturation. Proc Natl Acad Sci U S A. 2014;111(6):2301-6.

25. Yang L, Wei Y, Sun Y, Shi W, Yang J, Zhu L, et al. Interferon-gamma Inhibits Melanogenesis and Induces Apoptosis in Melanocytes: A Pivotal Role of CD8+ Cytotoxic T Lymphocytes in Vitiligo. Acta Derm Venereol. 2015;95(6):664-70.

26. Kholmanskikh O, van Baren N, Brasseur F, Ottaviani S, Vanacker J, Arts N, et al. Interleukins 1alpha and 1beta secreted by some melanoma cell lines strongly reduce expression of MITF-M and melanocyte differentiation antigens. Int J Cancer. 2010;127(7):1625-36.

27. Swope VB, Abdel-Malek Z, Kassem LM, Nordlund JJ. Interleukins 1 alpha and 6 and tumor necrosis factor-alpha are paracrine inhibitors of human melanocyte proliferation and melanogenesis. J Invest Dermatol. 1991;96(2):180-5.

28. Choi H, Choi H, Han J, Jin SH, Park JY, Shin DW, et al. IL-4 inhibits the melanogenesis of normal human melanocytes through the JAK2-STAT6 signaling pathway. J Invest Dermatol. 2013;133(2):528-36.

29. Kim DW, Jeon BJ, Hwang NH, Kim MS, Park SH, Dhong ES, et al. Adipose-derived stem cells inhibit epidermal melanocytes through an interleukin-6-mediated mechanism. Plast Reconstr Surg. 2014;134(3):470-80.

30. Hirobe T, Hasegawa K, Furuya R, Fujiwara R, Sato K. Effects of fibroblast-derived factors on the proliferation and differentiation of human melanocytes in culture. J Dermatol Sci. 2013;71(1):45-57.

31. Yamaguchi Y, Itami S, Watabe H, Yasumoto K, Abdel-Malek ZA, Kubo T, et al. Mesenchymal-epithelial interactions in the skin: increased expression of dickkopf1 by palmoplantar fibroblasts inhibits melanocyte growth and differentiation. J Cell Biol. 2004;165(2):275-85.

32. Hirobe T. Role of leukemia inhibitory factor in the regulation of the proliferation and differentiation of neonatal mouse epidermal melanocytes in culture. J Cell Physiol. 2002;192(3):315-26.

33. Busca R, Ballotti R. Cyclic AMP a key messenger in the regulation of skin pigmentation. Pigment Cell Res. 2000;13(2):60-9.

34. Mumm CD, Draznin M. Melanocortin-1 receptor: loss of function mutations and skin cancer. Dermatol Online J. 2006;12(5):13.

35. Passeron T, Valencia JC, Bertolotto C, Hoashi T, Le Pape E, Takahashi K, et al. SOX9 is a key player in ultraviolet B-induced melanocyte differentiation and pigmentation. Proc Natl Acad Sci U S A. 2007;104(35):13984-9.

36. Rousseau K, Kauser S, Pritchard LE, Warhurst A, Oliver RL, Slominski A, et al. Proopiomelanocortin (POMC), the ACTH/melanocortin precursor, is secreted by human epidermal keratinocytes and melanocytes and stimulates melanogenesis. FASEB J. 2007;21(8):1844-56.

37. Hunt G, Donatien PD, Lunec J, Todd C, Kyne S, Thody AJ. Cultured human melanocytes respond to MSH peptides and ACTH. Pigment Cell Res. 1994;7(4):217-21.

38. Suzuki I, Cone RD, Im S, Nordlund J, Abdel-Malek ZA. Binding of melanotropic hormones to the melanocortin receptor MC1R on human melanocytes stimulates proliferation and melanogenesis. Endocrinology. 1996;137(5):1627-33.

39. Aberdam E, Bertolotto C, Sviderskaya EV, de Thillot V, Hemesath TJ, Fisher DE, et al. Involvement of microphthalmia in the inhibition of melanocyte lineage differentiation and of melanogenesis by agouti signal protein. J Biol Chem. 1998;273(31):19560-5.

40. Spencer JD, Schallreuter KU. Regulation of pigmentation in human epidermal melanocytes by functional high-affinity beta-melanocyte-stimulating hormone/melanocortin-4 receptor signaling. Endocrinology. 2009;150(3):1250-8.

41. Imokawa G, Yada Y, Morisaki N, Kimura M. Biological characterization of human fibroblast-derived mitogenic factors for human melanocytes. Biochem J. 1998;330 ( Pt 3)(Pt 3):1235-9.

42. Matsumoto K, Tajima H, Nakamura T. Hepatocyte growth factor is a potent stimulator of human melanocyte DNA synthesis and growth. Biochem Biophys Res Commun. 1991;176(1):45-51.

43. Starner RJ, McClelland L, Abdel-Malek Z, Fricke A, Scott G. PGE(2) is a UVR-inducible autocrine factor for human melanocytes that stimulates tyrosinase activation. Exp Dermatol. 2010;19(7):682-4.

44. Scott G, Jacobs S, Leopardi S, Anthony FA, Learn D, Malaviya R, et al. Effects of PGF2alpha on human melanocytes and regulation of the FP receptor by ultraviolet radiation. Exp Cell Res. 2005;304(2):407-16.

45. Moustakas A. TGF-beta targets PAX3 to control melanocyte differentiation. Dev Cell. 2008;15(6):797-9.

46. Nishimura EK, Suzuki M, Igras V, Du J, Lonning S, Miyachi Y, et al. Key roles for transforming growth factor beta in melanocyte stem cell maintenance. Cell Stem Cell. 2010;6(2):130-40.

47. Singh M, Mansuri MS, Kadam A, Palit SP, Dwivedi M, Laddha NC, et al. Tumor Necrosis Factor-alpha affects melanocyte survival and melanin synthesis via multiple pathways in vitiligo. Cytokine. 2021;140:155432.
